# (2*R*,3*R*)-1-(4-Chloro­phen­yl)-2-[(*S*)-2-nitro-1-phenyl­eth­yl]-3-phenyl­pentan-1-one

**DOI:** 10.1107/S1600536811050306

**Published:** 2011-11-30

**Authors:** De-Long Duo, Cheng-Yan Ni, Qing-Song Wen

**Affiliations:** a School of Chemistry and Chemical Engineering, China West Normal University, Nanchong 637002, People’s Republic of China

## Abstract

The title compound, C_25_H_24_ClNO_3_, has three contiguous chiral centres. The absolute structure was determined by anomalous dispersion. The chloro­benzene ring is inclined to the two phenyl rings by 14.98 (9) and 59.05 (9)°. The two phenyl rings are inclined to one another by 49.51 (10)°. In the crystal, neighbouring mol­ecules are linked *via* C—H⋯O hydrogen bonds, forming chains propagating along [010]. There is also a C—H⋯π inter­action present that leads to the formation of a three-dimensional network.

## Related literature

For the synthesis of the title compound, see: Xu *et al.* (2007 ▶). For the role of pyrrolidine motifs as organo-catalysts in asymmetric catalysis, see: Taylor & Jacobsen (2006 ▶) and for their role in bioactive mol­ecules, see: Kawasaki *et al.* (2005 ▶).
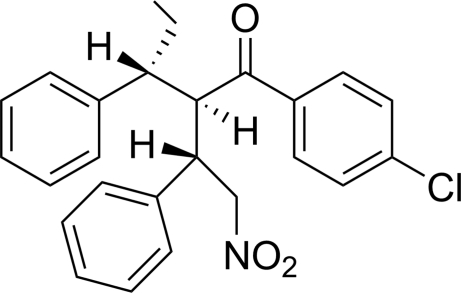


## Experimental

### 

#### Crystal data

C_25_H_24_ClNO_3_
*M* *_r_* = 421.90
Orthorhombic, 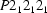

*a* = 8.4700 (1) Å
*b* = 13.1515 (2) Å
*c* = 20.7060 (2) Å
*V* = 2306.51 (5) Å^3^
*Z* = 4
Cu *K*α radiation
μ = 1.66 mm^−1^
*T* = 293 K
0.42 × 0.36 × 0.30 mm

#### Data collection

Gemini S Ultra Oxford Diffraction diffractometer
Absorption correction: multi-scan (*CrysAlis PRO*; Oxford Diffraction, 2009 ▶) *T*_min_ = 0.542, *T*_max_ = 0.635
22729 measured reflections
4292 independent reflections
4173 reflections with *I* > 2σ(*I*)
*R*_int_ = 0.019

#### Refinement

*R*[*F* ^2^ > 2σ(*F* ^2^)] = 0.035
*wR*(*F* ^2^) = 0.099
*S* = 1.04
4292 reflections
272 parameters
H-atom parameters constrained
Δρ_max_ = 0.14 e Å^−3^
Δρ_min_ = −0.25 e Å^−3^
Absolute structure: Flack (1983 ▶), 1824 Friedel pairs
Flack parameter: −0.010 (13)

### 

Data collection: *CrysAlis PRO* (Oxford Diffraction, 2009 ▶); cell refinement: *CrysAlis PRO*; data reduction: *CrysAlis PRO*; program(s) used to solve structure: *SHELXS97* (Sheldrick, 2008 ▶); program(s) used to refine structure: *SHELXL97* (Sheldrick, 2008 ▶); molecular graphics: *OLEX2* (Dolomanov *et al.*, 2009 ▶); software used to prepare material for publication: *OLEX2*.

## Supplementary Material

Crystal structure: contains datablock(s) global, I. DOI: 10.1107/S1600536811050306/su2340sup1.cif

Structure factors: contains datablock(s) I. DOI: 10.1107/S1600536811050306/su2340Isup2.hkl

Supplementary material file. DOI: 10.1107/S1600536811050306/su2340Isup3.cml

Additional supplementary materials:  crystallographic information; 3D view; checkCIF report

## Figures and Tables

**Table 1:** Hydrogen-bond geometry (Å, °) *CgA* is the centroid of the C1–C6 ring.

*D*—H⋯*A*	*D*—H	H⋯*A*	*D*⋯*A*	*D*—H⋯*A*
C5—H5⋯O3^i^	0.93	2.43	3.198 (5)	140
C12—H12⋯*CgA*^ii^	0.93	2.82	3.691 (4)	157

Symmetry codes: (i) 
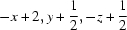
; (ii) 
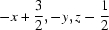
.

**Fig. 1.**
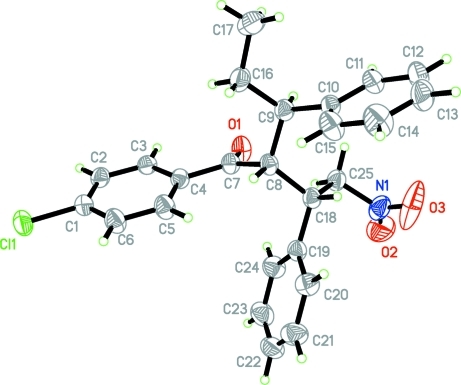
The molecular structure of the title compound, showing 30% probability displacement ellipsoids and the atomic numbering.

**Fig. 2.**
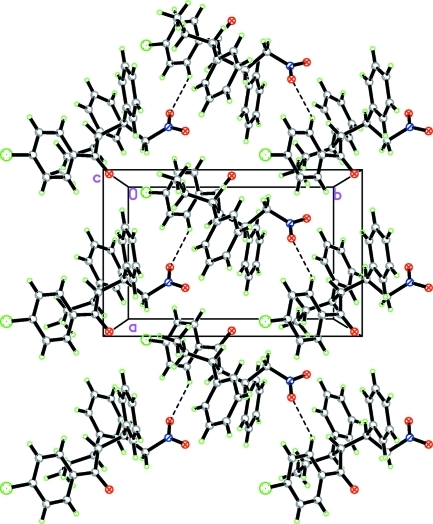
Crystal packing of the title compound, viewed along the c axis, showing the neighbouring molecules linked via C—H···O interactions (dashed lines), which generate chains propagating along the b axis direction.

## supplementary crystallographic information

### Comment

Chiral pyrrolidines are readily obtained using the corresponding γ-nitro substituted carbonyl compounds (Xu *et al.*, 2007). Pyrrolidine motifs are important as synthetic intermediates as well as organo-catalysts in asymmetric catalysis (Taylor & Jacobsen, 2006) and they are also present in many bioactive molecules (Kawasaki *et al.*, 2005). α,β-unsaturated ketones react with nitro-olfine derivatives to produce γ-nitro ketones in good yields with high diastereoselectivities and enantioselectivities. The crystal structure of one such compound, the title optically pure compound, is described herein.

In the title molecule (Fig. 1), carbon atoms C8, C9 and C18 are three contiguous chiral centres, *R*, *R*, S, respectively. The chlorobenzene ring, A (C1—C6), and phenyl ring B (C10—C15), are inclined to one another by 14.98 (9)°. Phenyl ring C (C19—C24) make dihedral angles with rings A and B of 59.05 (9) and 49.51 (10)°, respectively.

In the crystal, neighbouring molecules are linked *via* C—H···O hydrogen bonds to form chains which propagate along the *b* axis direction (Tabe 1 and Fig. 2). There is also a C—H···π interaction (Table 1) present which leads to the formation of a three-dimensional network.

### Experimental

The title compound was obtained by the procedure described by (Xu *et al.*, 2007). It was recrystallized from petroleun ether and ethyl acetate (*v*/*v* = 1:1), yielding colourless block-like crystals suitable for X-ray diffraction analysis.

### Refinement

H atoms were placed in calculated positions with C—H = 0.93–0.98 Å, and refined in riding mode with *U*_iso_(H) = 1.2*U*_eq_(C).

### Crystal data

**Table:**

C_25_H_24_ClNO_3_	*F*(000) = 888
*M**_r_* = 421.90	*D*_x_ = 1.215 Mg m^−^^3^
Orthorhombic, *P*2_1_2_1_2_1_	Cu *K*α radiation, λ = 1.54184 Å
Hall symbol: P 2ac 2ab	Cell parameters from 16354 reflections
*a* = 8.4700 (1) Å	θ = 3.4–69.8°
*b* = 13.1515 (2) Å	µ = 1.66 mm^−^^1^
*c* = 20.7060 (2) Å	*T* = 293 K
*V* = 2306.51 (5) Å^3^	Block, colourless
*Z* = 4	0.42 × 0.36 × 0.30 mm

### Data collection

**Table:**

Gemini S Ultra Oxford Diffraction diffractometer	4292 independent reflections
Radiation source: fine-focus sealed tube	4173 reflections with *I* > 2σ(*I*)
graphite	*R*_int_ = 0.019
Detector resolution: 15.9149 pixels mm^-1^	θ_max_ = 69.9°, θ_min_ = 4.0°
ω scans	*h* = −10→10
Absorption correction: multi-scan (*CrysAlis PRO*; Oxford Diffraction, 2009)	*k* = −15→16
*T*_min_ = 0.542, *T*_max_ = 0.635	*l* = −21→25
22729 measured reflections	

### Refinement

**Table:**

Refinement on *F*^2^	Secondary atom site location: difference Fourier map
Least-squares matrix: full	Hydrogen site location: inferred from neighbouring sites
*R*[*F*^2^ > 2σ(*F*^2^)] = 0.035	H-atom parameters constrained
*wR*(*F*^2^) = 0.099	*w* = 1/[σ^2^(*F*_o_^2^) + (0.0641*P*)^2^ + 0.1848*P*] where *P* = (*F*_o_^2^ + 2*F*_c_^2^)/3
*S* = 1.04	(Δ/σ)_max_ = 0.001
4292 reflections	Δρ_max_ = 0.14 e Å^−^^3^
272 parameters	Δρ_min_ = −0.25 e Å^−^^3^
0 restraints	Absolute structure: Flack (1983), 1824 Friedel pairs
Primary atom site location: structure-invariant direct methods	Flack parameter: −0.010 (13)

### Special details

**Table:**

Geometry. All e.s.d.'s (except the e.s.d. in the dihedral angle between two l.s. planes) are estimated using the full covariance matrix. The cell e.s.d.'s are taken into account individually in the estimation of e.s.d.'s in distances, angles and torsion angles; correlations between e.s.d.'s in cell parameters are only used when they are defined by crystal symmetry. An approximate (isotropic) treatment of cell e.s.d.'s is used for estimating e.s.d.'s involving l.s. planes.
Refinement. Refinement of *F*^2^ against ALL reflections. The weighted *R*-factor *wR* and goodness of fit *S* are based on *F*^2^, conventional *R*-factors *R* are based on *F*, with *F* set to zero for negative *F*^2^. The threshold expression of *F*^2^ > σ(*F*^2^) is used only for calculating *R*-factors(gt) *etc*. and is not relevant to the choice of reflections for refinement. *R*-factors based on *F*^2^ are statistically about twice as large as those based on *F*, and *R*- factors based on ALL data will be even larger.

### Fractional atomic coordinates and isotropic or equivalent isotropic displacement parameters (Å^2^)

**Table:**

	*x*	*y*	*z*	*U*_iso_*/*U*_eq_	
Cl1	0.58917 (6)	0.37368 (4)	0.50249 (2)	0.08473 (17)	
O1	0.50133 (14)	0.00173 (10)	0.28825 (7)	0.0700 (3)	
O2	0.7782 (3)	−0.30696 (14)	0.25735 (12)	0.1277 (8)	
O3	0.9069 (3)	−0.23945 (18)	0.18103 (18)	0.1769 (14)	
N1	0.8118 (2)	−0.23648 (13)	0.22259 (12)	0.0826 (5)	
C1	0.5978 (2)	0.28418 (13)	0.44074 (8)	0.0595 (4)	
C2	0.46287 (19)	0.23354 (13)	0.42310 (7)	0.0565 (4)	
H2	0.3673	0.2482	0.4431	0.068*	
C3	0.47066 (18)	0.16045 (12)	0.37513 (7)	0.0509 (3)	
H3	0.3801	0.1251	0.3634	0.061*	
C4	0.61302 (18)	0.13951 (11)	0.34438 (7)	0.0495 (3)	
C5	0.7473 (2)	0.19166 (15)	0.36286 (9)	0.0646 (4)	
H5	0.8430	0.1778	0.3427	0.078*	
C6	0.7404 (2)	0.26467 (16)	0.41126 (9)	0.0697 (5)	
H6	0.8308	0.2999	0.4236	0.084*	
C7	0.61293 (18)	0.05938 (12)	0.29275 (7)	0.0519 (3)	
C8	0.74862 (17)	0.05223 (12)	0.24430 (7)	0.0499 (3)	
H8	0.8271	0.1037	0.2561	0.060*	
C9	0.68287 (19)	0.08030 (12)	0.17641 (8)	0.0544 (3)	
H9	0.5912	0.0368	0.1681	0.065*	
C10	0.80251 (19)	0.06096 (12)	0.12331 (7)	0.0528 (3)	
C11	0.7672 (2)	−0.00341 (15)	0.07273 (8)	0.0664 (4)	
H11	0.6694	−0.0355	0.0720	0.080*	
C12	0.8725 (3)	−0.02151 (18)	0.02330 (10)	0.0846 (6)	
H12	0.8454	−0.0651	−0.0103	0.102*	
C13	1.0170 (3)	0.0248 (2)	0.02388 (11)	0.0923 (7)	
H13	1.0893	0.0123	−0.0090	0.111*	
C14	1.0545 (3)	0.0903 (2)	0.07361 (12)	0.0973 (7)	
H14	1.1523	0.1224	0.0740	0.117*	
C15	0.9477 (2)	0.10866 (18)	0.12305 (10)	0.0763 (5)	
H15	0.9740	0.1533	0.1562	0.092*	
C16	0.6259 (3)	0.19137 (16)	0.17528 (9)	0.0745 (5)	
H16A	0.5516	0.2017	0.2104	0.089*	
H16B	0.7154	0.2359	0.1826	0.089*	
C17	0.5474 (3)	0.2207 (2)	0.11228 (12)	0.0983 (7)	
H17A	0.4587	0.1769	0.1046	0.148*	
H17B	0.6218	0.2139	0.0776	0.148*	
H17C	0.5119	0.2899	0.1147	0.148*	
C18	0.83087 (17)	−0.05335 (12)	0.24718 (7)	0.0512 (3)	
H18	0.9132	−0.0522	0.2139	0.061*	
C19	0.91484 (19)	−0.07057 (11)	0.31084 (7)	0.0529 (3)	
C20	1.0760 (2)	−0.05423 (14)	0.31501 (9)	0.0652 (4)	
H20	1.1310	−0.0316	0.2789	0.078*	
C21	1.1568 (3)	−0.07107 (16)	0.37241 (12)	0.0809 (6)	
H21	1.2651	−0.0595	0.3743	0.097*	
C22	1.0794 (3)	−0.10408 (16)	0.42545 (10)	0.0813 (6)	
H22	1.1345	−0.1166	0.4635	0.098*	
C23	0.9185 (3)	−0.11912 (17)	0.42302 (9)	0.0825 (6)	
H23	0.8646	−0.1406	0.4597	0.099*	
C24	0.8368 (2)	−0.10225 (17)	0.36589 (9)	0.0702 (5)	
H24	0.7282	−0.1124	0.3647	0.084*	
C25	0.7198 (2)	−0.14079 (12)	0.22928 (9)	0.0595 (4)	
H25A	0.6401	−0.1489	0.2626	0.071*	
H25B	0.6667	−0.1255	0.1889	0.071*	

### Atomic displacement parameters (Å^2^)

**Table:**

	*U*^11^	*U*^22^	*U*^33^	*U*^12^	*U*^13^	*U*^23^
Cl1	0.0818 (3)	0.0920 (3)	0.0804 (3)	0.0158 (3)	−0.0070 (2)	−0.0388 (2)
O1	0.0573 (6)	0.0679 (7)	0.0848 (8)	−0.0145 (6)	0.0197 (6)	−0.0212 (6)
O2	0.173 (2)	0.0687 (10)	0.1411 (17)	0.0274 (12)	−0.0380 (16)	0.0015 (11)
O3	0.1117 (16)	0.1051 (15)	0.314 (4)	0.0056 (12)	0.093 (2)	−0.073 (2)
N1	0.0698 (10)	0.0618 (10)	0.1162 (14)	0.0089 (8)	−0.0152 (10)	−0.0261 (10)
C1	0.0637 (9)	0.0612 (9)	0.0537 (8)	0.0092 (8)	−0.0010 (7)	−0.0099 (7)
C2	0.0540 (8)	0.0620 (8)	0.0536 (8)	0.0105 (7)	0.0070 (6)	−0.0017 (7)
C3	0.0480 (7)	0.0555 (8)	0.0493 (7)	0.0025 (6)	0.0041 (6)	0.0031 (6)
C4	0.0502 (7)	0.0518 (7)	0.0465 (7)	0.0006 (6)	0.0057 (6)	−0.0005 (6)
C5	0.0518 (8)	0.0776 (11)	0.0645 (9)	−0.0060 (8)	0.0100 (7)	−0.0164 (8)
C6	0.0586 (9)	0.0806 (12)	0.0698 (10)	−0.0073 (9)	0.0011 (8)	−0.0215 (9)
C7	0.0490 (7)	0.0529 (7)	0.0539 (7)	−0.0023 (6)	0.0069 (6)	−0.0027 (6)
C8	0.0467 (7)	0.0538 (7)	0.0493 (7)	−0.0039 (6)	0.0071 (6)	−0.0063 (6)
C9	0.0505 (7)	0.0588 (8)	0.0540 (8)	0.0016 (6)	0.0059 (6)	−0.0044 (7)
C10	0.0554 (8)	0.0547 (8)	0.0481 (7)	0.0027 (7)	0.0048 (6)	−0.0021 (6)
C11	0.0731 (10)	0.0700 (10)	0.0561 (9)	−0.0023 (9)	−0.0004 (8)	−0.0106 (8)
C12	0.1079 (17)	0.0870 (13)	0.0590 (10)	0.0122 (12)	0.0094 (11)	−0.0191 (9)
C13	0.0968 (16)	0.1098 (17)	0.0703 (12)	0.0169 (13)	0.0332 (12)	−0.0050 (12)
C14	0.0749 (13)	0.129 (2)	0.0881 (14)	−0.0200 (13)	0.0292 (11)	−0.0019 (14)
C15	0.0699 (10)	0.0919 (14)	0.0670 (10)	−0.0222 (10)	0.0142 (9)	−0.0140 (10)
C16	0.0882 (13)	0.0710 (11)	0.0642 (10)	0.0241 (10)	0.0141 (9)	0.0008 (8)
C17	0.1010 (17)	0.1026 (17)	0.0914 (15)	0.0363 (14)	−0.0009 (13)	0.0153 (13)
C18	0.0470 (7)	0.0555 (8)	0.0511 (7)	−0.0004 (6)	0.0059 (6)	−0.0062 (6)
C19	0.0539 (8)	0.0496 (7)	0.0553 (8)	−0.0017 (6)	0.0004 (6)	−0.0061 (6)
C20	0.0575 (9)	0.0636 (9)	0.0745 (10)	−0.0104 (8)	−0.0033 (8)	0.0006 (8)
C21	0.0746 (12)	0.0727 (12)	0.0954 (15)	−0.0100 (10)	−0.0249 (11)	−0.0021 (11)
C22	0.1026 (15)	0.0691 (11)	0.0723 (11)	0.0028 (11)	−0.0278 (11)	−0.0100 (9)
C23	0.1092 (16)	0.0836 (13)	0.0548 (9)	0.0021 (13)	0.0032 (10)	0.0032 (9)
C24	0.0649 (10)	0.0858 (12)	0.0597 (9)	−0.0035 (9)	0.0063 (8)	0.0015 (9)
C25	0.0562 (8)	0.0541 (8)	0.0680 (9)	0.0028 (7)	−0.0036 (7)	−0.0117 (7)

### Geometric parameters (Å, °)

**Table:**

Cl1—C1	1.7393 (15)	C12—H12	0.9300
O1—C7	1.2153 (19)	C13—C14	1.379 (4)
O2—N1	1.208 (3)	C13—H13	0.9300
O3—N1	1.180 (3)	C14—C15	1.387 (3)
N1—C25	1.487 (2)	C14—H14	0.9300
C1—C2	1.372 (3)	C15—H15	0.9300
C1—C6	1.378 (3)	C16—C17	1.514 (3)
C2—C3	1.384 (2)	C16—H16A	0.9700
C2—H2	0.9300	C16—H16B	0.9700
C3—C4	1.391 (2)	C17—H17A	0.9600
C3—H3	0.9300	C17—H17B	0.9600
C4—C5	1.382 (2)	C17—H17C	0.9600
C4—C7	1.501 (2)	C18—C19	1.515 (2)
C5—C6	1.389 (3)	C18—C25	1.531 (2)
C5—H5	0.9300	C18—H18	0.9800
C6—H6	0.9300	C19—C24	1.382 (2)
C7—C8	1.5285 (19)	C19—C20	1.385 (2)
C8—C18	1.555 (2)	C20—C21	1.389 (3)
C8—C9	1.556 (2)	C20—H20	0.9300
C8—H8	0.9800	C21—C22	1.351 (3)
C9—C10	1.517 (2)	C21—H21	0.9300
C9—C16	1.539 (2)	C22—C23	1.378 (4)
C9—H9	0.9800	C22—H22	0.9300
C10—C11	1.379 (2)	C23—C24	1.388 (3)
C10—C15	1.381 (2)	C23—H23	0.9300
C11—C12	1.378 (3)	C24—H24	0.9300
C11—H11	0.9300	C25—H25A	0.9700
C12—C13	1.367 (4)	C25—H25B	0.9700
			
O3—N1—O2	124.8 (2)	C13—C14—C15	120.6 (2)
O3—N1—C25	117.0 (2)	C13—C14—H14	119.7
O2—N1—C25	118.1 (2)	C15—C14—H14	119.7
C2—C1—C6	121.45 (14)	C10—C15—C14	120.29 (19)
C2—C1—Cl1	119.29 (12)	C10—C15—H15	119.9
C6—C1—Cl1	119.25 (14)	C14—C15—H15	119.9
C1—C2—C3	119.24 (14)	C17—C16—C9	113.12 (18)
C1—C2—H2	120.4	C17—C16—H16A	109.0
C3—C2—H2	120.4	C9—C16—H16A	109.0
C2—C3—C4	120.49 (14)	C17—C16—H16B	109.0
C2—C3—H3	119.8	C9—C16—H16B	109.0
C4—C3—H3	119.8	H16A—C16—H16B	107.8
C5—C4—C3	119.23 (13)	C16—C17—H17A	109.5
C5—C4—C7	123.09 (13)	C16—C17—H17B	109.5
C3—C4—C7	117.67 (13)	H17A—C17—H17B	109.5
C4—C5—C6	120.54 (16)	C16—C17—H17C	109.5
C4—C5—H5	119.7	H17A—C17—H17C	109.5
C6—C5—H5	119.7	H17B—C17—H17C	109.5
C1—C6—C5	119.03 (17)	C19—C18—C25	112.75 (14)
C1—C6—H6	120.5	C19—C18—C8	112.17 (12)
C5—C6—H6	120.5	C25—C18—C8	112.71 (13)
O1—C7—C4	119.54 (13)	C19—C18—H18	106.2
O1—C7—C8	119.74 (13)	C25—C18—H18	106.2
C4—C7—C8	120.68 (13)	C8—C18—H18	106.2
C7—C8—C18	111.51 (12)	C24—C19—C20	117.82 (16)
C7—C8—C9	108.01 (12)	C24—C19—C18	122.57 (15)
C18—C8—C9	114.01 (12)	C20—C19—C18	119.61 (15)
C7—C8—H8	107.7	C19—C20—C21	120.96 (19)
C18—C8—H8	107.7	C19—C20—H20	119.5
C9—C8—H8	107.7	C21—C20—H20	119.5
C10—C9—C16	110.97 (14)	C22—C21—C20	120.5 (2)
C10—C9—C8	112.08 (12)	C22—C21—H21	119.7
C16—C9—C8	110.56 (14)	C20—C21—H21	119.7
C10—C9—H9	107.7	C21—C22—C23	119.76 (19)
C16—C9—H9	107.7	C21—C22—H22	120.1
C8—C9—H9	107.7	C23—C22—H22	120.1
C11—C10—C15	117.99 (16)	C22—C23—C24	120.1 (2)
C11—C10—C9	120.55 (15)	C22—C23—H23	120.0
C15—C10—C9	121.44 (15)	C24—C23—H23	120.0
C12—C11—C10	121.95 (19)	C19—C24—C23	120.83 (19)
C12—C11—H11	119.0	C19—C24—H24	119.6
C10—C11—H11	119.0	C23—C24—H24	119.6
C13—C12—C11	119.7 (2)	N1—C25—C18	109.64 (14)
C13—C12—H12	120.1	N1—C25—H25A	109.7
C11—C12—H12	120.1	C18—C25—H25A	109.7
C12—C13—C14	119.41 (19)	N1—C25—H25B	109.7
C12—C13—H13	120.3	C18—C25—H25B	109.7
C14—C13—H13	120.3	H25A—C25—H25B	108.2
			
C6—C1—C2—C3	0.7 (3)	C10—C11—C12—C13	0.3 (4)
Cl1—C1—C2—C3	−178.32 (12)	C11—C12—C13—C14	−0.9 (4)
C1—C2—C3—C4	−1.0 (2)	C12—C13—C14—C15	0.5 (4)
C2—C3—C4—C5	0.8 (2)	C11—C10—C15—C14	−1.0 (3)
C2—C3—C4—C7	−179.95 (14)	C9—C10—C15—C14	−179.4 (2)
C3—C4—C5—C6	−0.4 (3)	C13—C14—C15—C10	0.4 (4)
C7—C4—C5—C6	−179.60 (17)	C10—C9—C16—C17	60.0 (2)
C2—C1—C6—C5	−0.3 (3)	C8—C9—C16—C17	−174.97 (18)
Cl1—C1—C6—C5	178.72 (16)	C7—C8—C18—C19	−66.22 (16)
C4—C5—C6—C1	0.2 (3)	C9—C8—C18—C19	171.13 (12)
C5—C4—C7—O1	164.28 (18)	C7—C8—C18—C25	62.33 (17)
C3—C4—C7—O1	−14.9 (2)	C9—C8—C18—C25	−60.31 (17)
C5—C4—C7—C8	−18.1 (2)	C25—C18—C19—C24	−47.1 (2)
C3—C4—C7—C8	162.74 (14)	C8—C18—C19—C24	81.41 (19)
O1—C7—C8—C18	−61.3 (2)	C25—C18—C19—C20	132.96 (16)
C4—C7—C8—C18	121.03 (15)	C8—C18—C19—C20	−98.51 (16)
O1—C7—C8—C9	64.69 (19)	C24—C19—C20—C21	1.2 (3)
C4—C7—C8—C9	−112.95 (15)	C18—C19—C20—C21	−178.86 (17)
C7—C8—C9—C10	−171.81 (13)	C19—C20—C21—C22	0.1 (3)
C18—C8—C9—C10	−47.28 (18)	C20—C21—C22—C23	−1.4 (3)
C7—C8—C9—C16	63.81 (17)	C21—C22—C23—C24	1.2 (3)
C18—C8—C9—C16	−171.65 (14)	C20—C19—C24—C23	−1.4 (3)
C16—C9—C10—C11	−113.53 (19)	C18—C19—C24—C23	178.72 (17)
C8—C9—C10—C11	122.32 (17)	C22—C23—C24—C19	0.2 (3)
C16—C9—C10—C15	64.8 (2)	O3—N1—C25—C18	−63.7 (3)
C8—C9—C10—C15	−59.3 (2)	O2—N1—C25—C18	120.2 (2)
C15—C10—C11—C12	0.7 (3)	C19—C18—C25—N1	−60.96 (19)
C9—C10—C11—C12	179.12 (18)	C8—C18—C25—N1	170.79 (16)

### Hydrogen-bond geometry (Å, °)

**Table:**

CgA is the centroid of the C1–C6 ring.

**Table:**

*D*—H···*A*	*D*—H	H···*A*	*D*···*A*	*D*—H···*A*
C5—H5···O3^i^	0.93	2.43	3.198 (5)	140
C12—H12···CgA^ii^	0.93	2.82	3.691 (4)	157

Symmetry codes: (i) −*x*+2, *y*+1/2, −*z*+1/2; (ii) −*x*+3/2, −*y*, *z*−1/2.
